# Supplementary material for: Content-rich biological network constructed by mining PubMed abstracts
Source: BMC Bioinformatics. 2004 Oct 8;5:147. doi: 10.1186/1471-2105-5-147 (PMC528731; doi:10.1186/1471-2105-5-147)
Supplement: Additional File 2 — The original results of the above study (non-essential files are deleted to keep the file size under the limit set by BMC bioinformatics). [file 1471-2105-5-147-S2.bz2 › chilibotAdditionalFile2/dip05/497ID10187774E10660/html/TGFB1_ACVRL1.html]

 


 **TGFB1** and **ACVRL1** 
  
Found 99 abstracts in PubMed, retrieved 05.  
 

 What does Google say? 
 PDF only 
| .edu only 

---

**Interactive relationship** (e.g. stimulation, inhibition, etc)

**Stimulatory relationship**- Immunohistochemistry demonstrated that, compared to controls, shunt lambs have increased TGF beta1  [ **TGFB1** ]  and TbetaRI expression in the smooth muscle layer, and increased TGF beta1  [ **TGFB1** ]  and ALK 1  [ **ACVRL1** ]  in the endothelium of small pulmonary arteries < ; 200 um at 1 and 4 weeks of age.  Ref: 12665463 Am J Physiol Lung Cell Mol Physiol< MedlineTA>Am J Physiol Lung Cell Mol Physiol,
**Neutral relationship**- Recent studies have identified mutations of the bone morphogenetic protein receptor 2 BMPR2 gene and the activin receptor like kinase 1  [ **ACVRL1** ]  ALK1  [ **ACVRL1** ]  gene, that affect the transforming growth factor beta TGF beta  [ **TGFB1** ]  receptor superfamily, a group of transmembrane signaling molecules with serine threonine kinase activity that are involved in the regulation of cell growth.  Ref: 12624796 Z Rheumatol, 2003
- Activin receptor like kinase 1  [ **ACVRL1** ]  ALK 1  [ **ACVRL1** ] , the gene mutated in HHT  [ **ACVRL1** ]  type 2 HHT2  [ **ACVRL1** ] , is a serine threonine kinase receptor type I of the TGF beta  [ **TGFB1** ]  superfamily, specifically expressed on endothelial cells.  Ref: 12700602 Eur J Hum Genet, 2003

**Non-interactive relationship** (e.g. studied together, co-existance, homology, etc.)

- TGF beta1  [ **TGFB1** ] , ALK 1  [ **ACVRL1** ] , ALK  [ **ACVRL1** ]  5, and TbetaRII were also detected by immunohistochemistry.  Ref: 12883979 Pediatr NephrolPediatr Nephrol, 2003
- Western blots demonstrated that TGF beta1  [ **TGFB1** ]  and ALK 1  [ **ACVRL1** ]  expression were elevated in the shunt as compared to control lambs at 1 and 4 weeks of age P< ; 0.05.  Ref: 12665463 Am J Physiol Lung Cell Mol Physiol< MedlineTA>Am J Physiol Lung Cell Mol Physiol, 2003
- To determine the potential role of TGF beta1  [ **TGFB1** ]  in this vascular remodeling, we then investigated vascular changes in expression and localization of TGF beta1  [ **TGFB1** ]  and its receptors TbetaRI, ALK 1  [ **ACVRL1** ] , and TbetaRII in the lungs of shunted and age matched control lambs at 1 day,, and 8 weeks of life.  Ref: 12665463 Am J Physiol Lung Cell Mol Physiol< MedlineTA>Am J Physiol Lung Cell Mol Physiol, 2003
- These results indicate that ACE inhibition in the developing kidney decreases TGF beta1  [ **TGFB1** ] , ALK 1  [ **ACVRL1** ] , and ALK  [ **ACVRL1** ]  5 expression, which may account for renal growth impairment.  Ref: 12883979 Pediatr NephrolPediatr Nephrol, 2003
- Newborn rat pups were treated with enalapril 30 mg kg per day or vehicle for 7 days, and kidneys were removed for Western blotting of TGF beta1  [ **TGFB1** ] , ALK 1  [ **ACVRL1** ] , ALK  [ **ACVRL1** ]  5, and TbetaRII, and for RT PCR of ALK  [ **ACVRL1** ]  5 and TbetaRII.  Ref: 12883979 Pediatr NephrolPediatr Nephrol, 2003
- This study was designed to investigate the relationship between renal growth impairment and the expression of transforming growth factor beta1  [ **TGFB1** ]  TGF beta1  [ **TGFB1** ] , TGF beta  [ **TGFB1** ]  receptor I TbetaRI, activin like kinase ALK 1  [ **ACVRL1** ]  and ALK  [ **ACVRL1** ]  5, and TGF beta  [ **TGFB1** ]  receptor II TbetaRII.  Ref: 12883979 Pediatr NephrolPediatr Nephrol, 2003
- Enalapril decreased renal TGF beta1  [ **TGFB1** ] , ALK 1  [ **ACVRL1** ] , and ALK  [ **ACVRL1** ]  5 protein expression P< ; 0.05.  Ref: 12883979 Pediatr NephrolPediatr Nephrol, 2003
